# Endoscopic mucosal resection with circumferential incision for treatment of rectal carcinoid tumours

**DOI:** 10.1186/1477-7819-12-23

**Published:** 2014-01-28

**Authors:** Jin Huang, Zhong-Sheng Lu, Yun-sheng Yang, Jing Yuan, Xiang-dong Wang, Jiang-yun Meng, Hong Du, Hong-bin Wang

**Affiliations:** 1 Department of Gastroenterology, Chinese PLA 153 Central Hospital, 602 Zhengshang Road, Zhengzhou, Henan Province 450000, China; 2 Department of Gastroenterology, Chinese PLA General Hospital, 28 Fuxing Road, Beijing 100853, China; 3 Department of Pathology, Chinese PLA General Hospital, Beijing, China; 4 Department of Gastroenterology, Chinese PLA General Hospital, Beijing, China

**Keywords:** CIEMR, Endoscopic mucosal resection (EMR), Rectal carcinoid tumours

## Abstract

**Background:**

Endoscopic mucosal resection (EMR) is simple and quick and has low complication rates. However, the disadvantage of local recurrence or remnant rate limits the use of this technique. We aimed to analyse the outcomes of conventional EMR and EMR with circumferential incision (CIEMR), a simplified modification of EMR, in the endoscopic treatment of rectal carcinoid tumours.

**Methods:**

A total of 59 consecutive patients with rectal carcinoid tumours without regional lymph node enlargement confirmed by endoscopic ultrasonography were included in the study. These patients underwent endoscopic treatment from January 2009 to September 2011 and were randomly designated into CIEMR (*n* = 31) or EMR group (*n* = 28). *En bloc* resection rate, pathological complete resection rate, procedure time, complications and follow-up outcomes were analysed.

**Results:**

The *en bloc* resection rate was not significantly different between the CIEMR and EMR groups (100% versus 96.55%, *P* > 0.05). The pathological complete resection rate was higher in the CIEMR group than in the EMR group (96.7% versus 82.14%, *P* < 0.05). The overall complication rate, delayed bleeding and procedure time were not significantly different between the two groups. No recurrence was observed in either the EMR or CIEMR group.

**Conclusions:**

CIEMR optimises the procedure of EMR and simplifies the technique of endoscopic submucosal dissection; thus, it has a better histologically complete resection rate and more acceptable complication rate than EMR. Thus, CIEMR may be preferable to conventional EMR for resection of rectal carcinoid tumours less than 15 mm.

## Background

Carcinoid tumour is a slow-growing type of neuro-endocrine tumour that originates in the cells of the neuro-endocrine system [1]. Over two-thirds of carcinoid tumours are found in the gastrointestinal tract [2]. Rectal carcinoid tumours are uncommon, representing 1.1% to 1.3% of all rectal neoplasms [3,4]. Most rectal carcinoid tumours are asymptomatic and found during screening colonoscopy. The surgical treatment for carcinoid tumours is generally dictated to a degree, but not absolutely, by size [5]. Tumours less than 1 cm in diameter are rarely associated with metastatic disease. Tumours measuring from 1.0 cm to 1.9 cm in diameter tend to show a metastatic rate of 4% to 30% [6,7]. Lymph node or liver metastases are observed in over 80% of tumours measuring more than 2 cm in diameter. Therefore, small rectal carcinoid tumours are usually managed with local excision including endoscopic techniques [8,9].

Endoscopic mucosal resection (EMR) is generally performed for gastrointestinal lesions because of its simplicity, quick operation and low complication rates [10-13]. However, locally recurrent or remnant tumours after EMR of gastrointestinal tumours have been increasingly reported [14,15], coinciding with increased use of EMR. EMR could be used to complete resection of mucosal lesions less than 2 cm, and rectal carcinoid tumours suitable for endoscopic treatment are generally less than 2 cm, but lateral and vertical margin involvement has recently been reported [16]. Endoscopic submucosal dissection (ESD) is a therapeutic technique used to treat gastrointestinal neoplasms with a high *en bloc* resection rate. However, ESD is not widely used to treat rectal carcinoid tumours in China because of its technical difficulty, longer procedure time and increased risk of perforation. Circumferential incision is one of the procedures of ESD. Hirao *et al*. [17] first reported EMR with circumferential incision (CIEMR) in 1986. The application of CIEMR in early gastric cancer has been previously reported [18,19]. CIEMR reduced the operative time and avoided the risk of perforation for lesions less than 2 cm.

The present study compared CIEMR with EMR for the treatment of rectal carcinoid tumours. *En bloc* resection rate, histological complete resection rate, complications, resection time and follow-up outcomes were measured to determine an effective and simple treatment approach for rectal carcinoid tumours. The aim of this study was to assess the outcomes of conventional EMR and CIEMR in the endoscopic treatment of rectal carcinoid tumours.

## Methods

### Patients

Conventional EMR and CIEMR were performed on 59 lesions in 59 patients with rectal carcinoid tumours. These treatments were carried out at the Chinese PLA General Hospital, Beijing, China between January 2009 and September 2011. The patients were randomly divided into CIEMR (*n* = 31) or EMR groups (*n* = 28). All patients were asymptomatic, and all tumours were found incidentally on screening colonoscopy. The inclusion criterion was patients with rectal carcinoid tumours less than 15 mm without significant evidence of regional lymph node enlargement on computer tomography (CT) scanning or endoscopic ultrasonography (EUS). All lesions were diagnosed by EUS as tumours in the submucous layer and confirmed by histological evaluation of endoscopic biopsy specimens before EMR/CIEMR. Rectal carcinoid tumours with distant metastases to the liver or lung on CT scans were excluded. Two experienced endoscopists (LZS and HJ) performed all EMR/CIEMR procedures. Informed consent was obtained from each patient. All aspects of this study were approved by the medical ethical committee of the Chinese PLA General Hospital.

The endpoint of the present study was the differences of clinical efficacy between CIEMR and EMR, the variables evaluated included *en bloc* resection rate and pathological complete resection rate, as well as procedure time, complications and follow-up outcomes.

### CIEMR and conventional EMR procedures

A single-accessory channel endoscope (GIF-Q260J; Olympus, Tokyo, Japan) was used during the procedure. A short transparent cap was attached to the tip of the endoscope (ND-201-11802, Olympus, Tokyo, Japan). An electrosurgical current generator (VIO200, ERBE, Tubingen, Germany) was used for endoscopic treatment.

**Figure 1:**
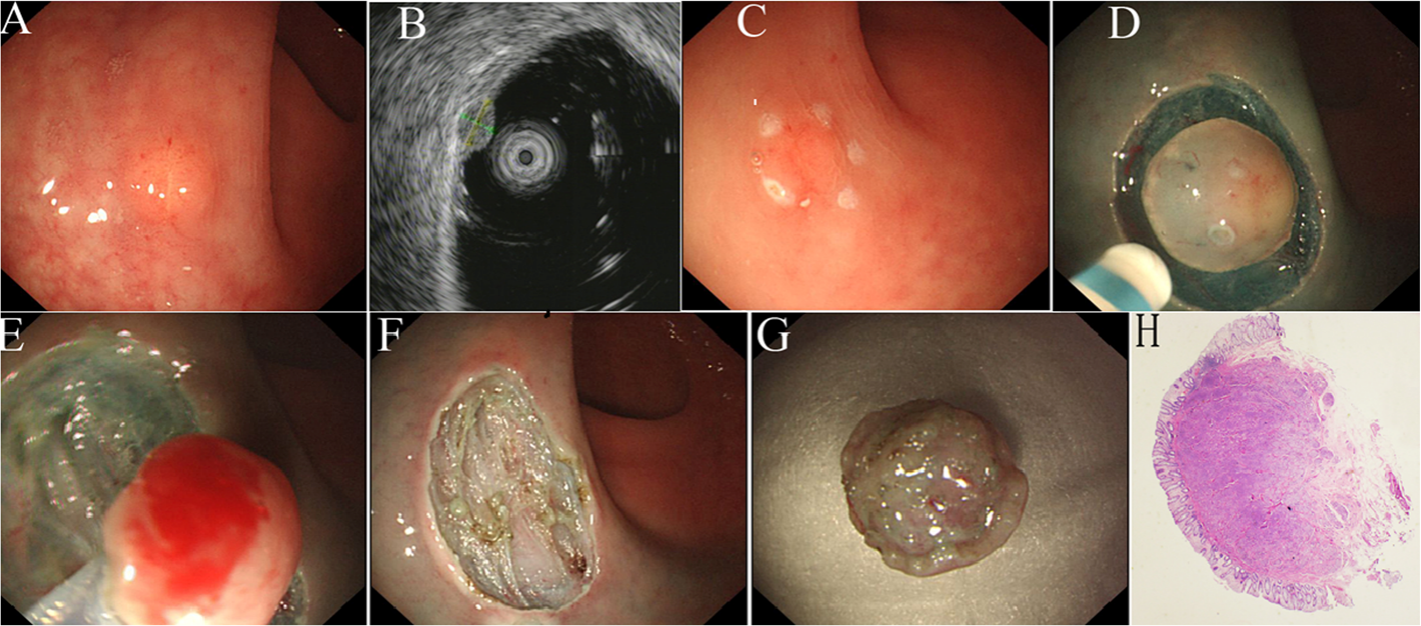
**Endoscopic mucosal resection with circumferential incision of a rectal carcinoid tumour. (A)** Conventional endoscopic view showing a carcinoid tumour in the rectum; **(B)** Endoscopic ultrasonography (EUS) image showing the lesion not to invade the muscularis propria layer; **(C)** Marking dots made around the lesion; **(D)** Circumferential incision around the dots; **(E)** Snaring after circumferential incision; **(F)** Region after endoscopic mucosal resection with circumferential incision; **(G)** Resected specimen; **(H)** Histologic view of a rectal carcinoid tumor obtained by endoscopic submucosal dissection (ESD) (H&E stain; ×40).

CIEMR was performed as follows (Figure 1): 1) marking dots were made approximately 2 mm to 3 mm outside the lesion with a dual knife (KD-650Q, Olympus, Tokyo, Japan); 2) a solution (250 mL fructose-glycerol (Chia Tai Fenghai Pharmaceutical Co., JS, China) + 1 mL epinephrine + 0.3 mL methylthioninium chloride) was submucosally injected around the lesion using a needle (INJ1-A1-07-5-23-180, Medwork, Neuss, Germany). After the injection, only the mucosa was lifted off the muscularis propria layer; 3) then, the mucosa was incised outside the marker dots using a dual knife; 4) the tumour was resected with a snare. The visibly exposed vessels on the artificial ulcer were coagulated with a Coagrasper (FD-410LR, Olympus, Tokyo, Japan) or argon plasma coagulation to prevent delayed bleeding. CIEMR resection time was counted from the time of making the marking dots to the completion of resection.

Conventional EMR procedure in this study was performed by ER-cap technique. An ER kit (Olympus, Tokyo, Japan) was used, which contains a spraying catheter, an injection needle, a hard oblique cap (inner 12 mm) and a crescent-shaped snare. The cap was attached to the tip of the endoscope, and the endoscope was reintroduced. The lesion was initially lifted by submucosal injection. Then, the snare was prelooped in the distal rim of the cap, and the mucosa was sucked into the cap before the snare was tightened. Resection time of conventional EMR was defined as the time from the submucosal injection to the completion of resection.

### Histopathological evaluation

Specimens were microscopically examined for histopathological type, invasion depth, lateral and vertical resection margins and lymphovascular involvement. *En bloc* resection referred to resection of the entire tumour in one piece rather than piecemeal. The histopathologic evaluation of gastrointestinal neuro-endocrine neoplasm in this study was NET - G1 according to the World Health Organization (WHO) 2010 classification of tumors of the digestive system.

A pathological complete resection was defined as an *en bloc* resection with no lateral and vertical margin involvement of the resected specimen and no lymphovascular invasion.

### Follow-up

The patients were followed-up by colonoscopy at 3, 6 and 12 months and then annually thereafter. The patients with vertical or lateral resection margin involvement were recommended for additional surgery and followed-up with chest radiography and abdominal-pelvic CT scanning.

### Statistical analyses

Statistical analysis was performed by using SPSS software (version 12.0; SPSS Inc, Chicago, IL, USA). Significant difference between the two groups was analyzed using Fisher exact test and Mann–Whitney *U*-test. *P* < 0.05 was considered significant for two-tailed tests.

## Results

### Characteristics of patients and tumours

Sex and age distribution were similar in conventional EMR and CIEMR groups (31 CIEMR and 28 EMR). The endoscopically estimated diameter of rectal carcinoid tumours was 3 mm to 15 mm in both groups. The histologically measured sizes of the resected specimens were similar to the endoscopically estimated diameters. Table 1 shows the characteristics of the two groups.

**Table 1:** Characteristics of patients and tumours

	**CIEMR**	**EMR**	***P*-value**
Age, mean ± SD, years	50 ± 10.1	49 ± 12.2	0.62
Sex, (n%)			0.75
Male	17	15	
Female	14	13	
Tumor size (mm)^a^			0.73
Mean ± SD	9 ± 2.5	8 ± 3.3	
Range	3 to 15	3 to 15	
Distance from the anal verge (cm)	6.1 ± 2.5	6.2 ± 3.0	0.67

CIEMR, conventional endoscopic mucosal resection with circumferential incision.

EMR, endoscopic mucosal resection.

^a^Tumour size was determined by pathological findings.

### Outcomes of CIEMR/EMR

**Figure 2:**
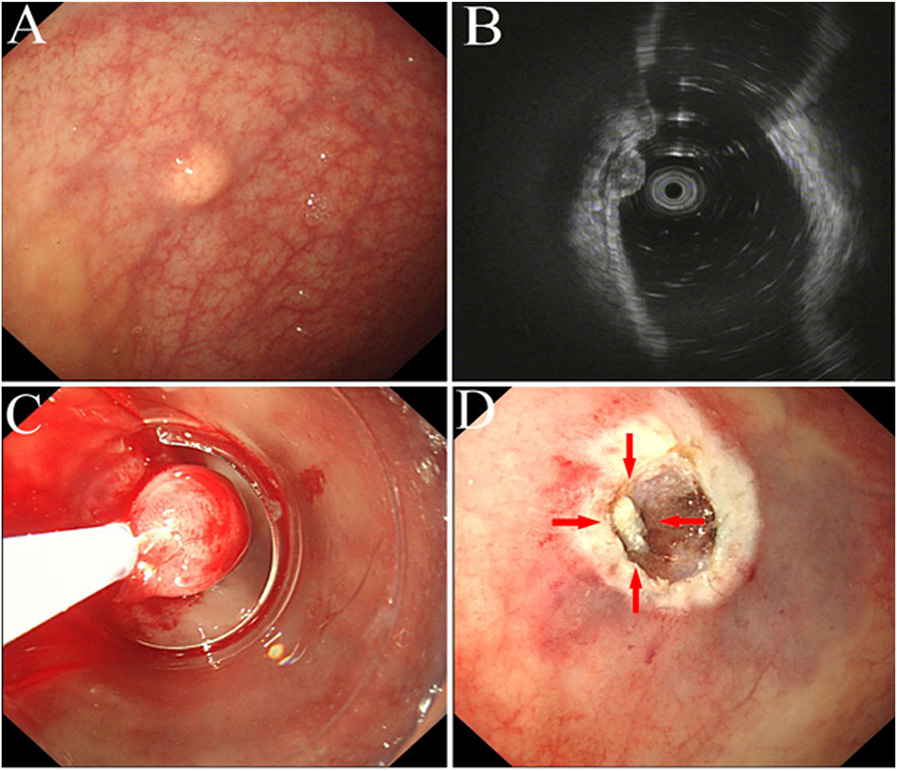
**Incomplete resection using endoscopic mucosal resection (EMR). (A)** Conventional endoscopic view showing a carcinoid tumour in the rectum; **(B)** Endoscopic ultrasonography (EUS) image showing the lesion in the muscularis propria layer; **(C)** Snaring after submucosal injection; **(D)** Remnant carcinoid tumour on post-EMR ulcer (red arrow).

The clinical outcomes of the CIEMR and EMR groups are shown in Table 2. The *en bloc* resection rate was not significantly different between the two groups (*P* > 0.05) (Figure 2). The pathological complete resection rate was higher in the CIEMR group than in the EMR group (*P* < 0.05). Lateral resection margin involvement was observed in three cases (10.3%) in the EMR group but not in the CIEMR group. Vertical resection margin involvement was observed in two cases (6.9%) in the EMR group versus one in the CIEMR group (3.3%). No lymphovascular invasion occurred in either the CIEMR or EMR group. The median CIEMR operative time and EMR operative time were 7.6 minutes (range, 5 minutes to 13 minutes) and 4.2 minutes (range, 2 minutes to 10 minutes), respectively.

**Table 2:** Clinical outcomes of circumferential incision endoscopic mucosal resection (CIEMR) and endoscopic mucosal resection (EMR)

	**CIEMR**	**EMR**	***P*-value**
*En bloc* resection, n (%)	100	96.55	0.747
Pathologic complete resection, n (%)	96.7	82.14	0.043
Resection time (minutes)	7.6	4.2	0.028
Complication, n (%)			
Delayed bleeding	0	0	1
Perforation	0	0	1
Follow-up	19.1(8.6)	18.3(7.9)	0.588

CIEMR, endoscopic mucosal resection with circumferential incision.

EMR, endoscopic mucosal resection.

### Complications

No perforations or delayed bleeding occurred in any xpatient of both groups. Procedure-related bleeding occurred in all CIEMR cases and in 21 of 28 of EMR cases, but haemostasis was achieved by endoscopic treatment. None of the patients required blood transfusion. The complication rates did not significantly differ between the EMR and CIEMR groups (*P* = 1.00).

### Follow-up outcomes

No local recurrence occurred during a median follow-up period of 20 months (range, 3 months to 35 months) in patients with pathological complete resection in both the CIEMR and EMR groups. An incomplete pathological resection was found in five patients of the EMR group. Six patients with vertical and lateral resection margin involvement were converted to open surgery. Postoperative pathological diagnosis was positive for carcinoid in two patients in the EMR groups. No local remnant lesion was observed during the median follow-up period of 17 months (range, 3 months to 30 months).

## Discussion

EMR is a safe and useful endoscopic strategy for small and superficial neoplasms confined to the mucosa or superficial submucosa in the colorectum. However, the high recurrence rate of residual lesions is a restrictive factor for EMR application. ESD, devoid of the disadvantages of EMR, is performed to locally treat small rectal carcinoid tumours to achieve complete resection rates, but the entire ESD is difficult and the complication rate is high. This study performed CIEMR in rectal carcinoid tumours and compared the clinical outcomes between CIEMR and EMR.

Patients with rectal carcinoid tumours not larger than 15 mm were chosen in this study. Given that most rectal carcinoid tumours that we detected were small, the result agreed well with that reported in the literature [20]. Metastasis is reported to occur in 5% to 15% of carcinoid tumours measuring 10 mm to 19 mm, and the frequency increases to over 80% for tumours sized 20 mm or larger [21]. ESD was performed in patients with rectal carcinoid tumours larger than 15 mm in our hospital. The *en bloc* resection rate was not significantly different between the CIEMR group and the EMR group because the lesion size was small. CIEMR is a modification on EMR that involves the circumferential incision procedure of ESD to ensure a safe margin. Lateral resection margin involvement was observed in three cases in the EMR group versus none in the CIEMR group. Although the procedure time of CIEMR seemed significantly longer than that of EMR (7.6 minutes versus 4.2 minutes) because of marking, circumferential resection and other extra steps, the mean resection time (7.6 minutes) was acceptable. In this study, the complication rate in the CIEMR group was similar to that in the EMR group. No perforation occurred in either group, probably because the lesions were small. Intraoperative bleeding was successfully managed by endoscopic treatment, and no delayed bleeding occurred.

Compared with conventional EMR, the advantage of CIEMR is risk reduction of residual tumours because the side margin of the lesion is clearly exposed after circumferential incision. Given that residual or recurrent tumours usually develop from remnant tumour tissues from the resected margin, determining the lateral margin blindly and confirming the presence or absence of any residual tumour after resection because of ablation are difficult for conventional EMR. After circumferential incision, the snare can be easily placed along the incision, and the tumour margin can be reliably cut. But the disadvantage of CIEMR is the longer procedure time versus EMR. Compared with ESD, CIEMR procedure is easier because submucosal dissection, one of the most difficult ESD procedures, is omitted. Snaring becomes simple and safe after circumferential incision. And the procedure time of CIEMR is shortened and the risk of complications such as perforation is reduced.

The first limitation of this study is the single-centre design. More experience from other centres is necessary to define the indication for CIEMR. Second, CIEMR or EMR was only performed in rectal carcinoid tumours. Considering that the rectum is wide and fixed and has fewer mucosal folds, endoscopic manoeuvrability in the rectal area is usually better than that in other locations of the gastrointestinal tract. Third, the follow-up period was not sufficiently long. Finally, outcomes after CIEMR and EMR were analysed in this research, but ESD has been found feasible for the treatment of rectal carcinoid tumours in many studies [16,22-24]. In the future, we will compare ESD with CIEMR for the endoscopic treatment of rectal carcinoid tumours.

## Conclusion

In conclusion, CIEMR optimises the procedure of EMR and simplifies the technique of ESD. This method shows better histologically complete resection rate and more acceptable complication rate than EMR. CIEMR is a good alternative to EMR for *en bloc* resection of rectal carcinoid tumours less than 15 mm in diameter.

## Abbreviations

EMR: endoscopic mucosal resection; CIEMR: endoscopic mucosal resection with circumferential incision; ESD: endsocopic submucosal dissection; CT: computer tomography; EUS: endoscopic ultrasonography; WHO: World Health Organization.

## Competing interest

The authors declare that they have no competing interests.
